# The relationship between triglyceride to high-density lipoprotein cholesterol ratio and cardiovascular high risk: a cross-sectional investigation

**DOI:** 10.3389/fendo.2025.1688624

**Published:** 2025-11-27

**Authors:** Pengcheng Li, Jirui Cai, Shuaifang Yuan, Yapeng Li, Huiting Shi, Cui Liang, Bing He, Qiaotao Xie, Baocang Lei, Jing Bai, Nan Wang, Dongliang Liu, Qichao Wang, Jianwei Xiong, Jin Wang, Haoran Wang

**Affiliations:** 1 Department of Cardiology, The First Affiliated Hospital of Zhengzhou University, Zhengzhou, China; 2 Luohe Central Hospital, Luohe Medical College, Luohe, China

**Keywords:** TG/HDL-C ratio, cardiovascular high risk, cross-sectional study, public health, lipid fractions

## Abstract

**Aims:**

The triglyceride to high-density lipoprotein cholesterol (TG/HDL-C) ratio reflects the balance between atherogenic and anti-atherogenic lipid fractions. The study aims to investigate the utility of the TG/HDL-C ratio in identifying high-risk groups for cardiovascular disease (CVD) within the general population.

**Methods:**

The current study was a branch of the ChinaHEART cohort in middle China that involved a total of 6,593 community-dwelling adults. We examine the association between TG/HDL-C and CVD high-risk in a cross-sectional investigation.

**Results:**

Results from restricted cubic spline and ROC analyses revealed a significant relationship between higher TG/HDL-C levels and increased odds of CVD high-risk, with the ratio exhibiting good discriminatory power (AUC = 0.820 in the fully adjusted model). Logistic regression further supported this association, indicating a 1.21-fold rise in the odds of high risk for each unit increase in TG/HDL-C.

**Conclusions:**

The TG/HDL ratio shows promise for CVD risk assessment. Incorporating the TG/HDL ratio into risk assessment models may provide supplementary information to healthcare professionals for risk stratification.

## Introduction

Cardiovascular diseases (CVDs) remain the leading cause of morbidity and mortality worldwide, posing a substantial global health and economic burden. Early identification of individuals at high risk of developing CVD is crucial for implementing targeted prevention and management strategies (1). Traditional risk factors such as hypertension, dyslipidemia, smoking, and diabetes have been widely recognized, but there is a growing interest in exploring novel biomarkers that may enhance risk stratification in the general population (2).

In recent years, the triglyceride to high-density lipoprotein cholesterol (TG/HDL-C) ratio has emerged as a potential marker for cardiovascular risk assessment (3–7). This ratio reflects the balance between atherogenic and anti-atherogenic lipid fractions and has been proposed as a simple and cost-effective tool to identify individuals at increased risk of CVD. Some studies have found that this indicator is associated with morbidity and mortality from CVD (3, 8). However, its precise utility in identifying high-risk groups for CVD within the general population, particularly using established risk assessment frameworks, remains underexplored.

Our study aims to address this gap in knowledge by conducting a cross-sectional observational study to evaluate the utility of the TG/HDL-C ratio in identifying high-risk individuals for CVD in the general population. By examining this novel biomarker in a large and diverse sample, we seek to elucidate its potential as a screening tool for early risk stratification and intervention. If we can effectively identify individuals at high risk of CVD in the general population using this indicator, we can intervene at earlier stages of CVD, thereby maximizing the prevention of major adverse cardiovascular events.

## Methods

### Study population and data collection

The current study is a branch of the China Health Evaluation and Risk Reduction through nationwide Teamwork (ChinaHEART) in Luohe City, involving 6,860 community-dwelling adults recruited from two township hospitals in Luohe City between 2 November 2021 and 20 February 2022. The ChinaHEART cohort is a population cohort launched by the China National Center for Cardiovascular Diseases (NCCD) initially aiming at CVD risk screening and management (9). Study methods have been described previously (10). The study strictly adhered to ethical guidelines, with approval granted by the Ethics Committee of Fuwai Hospital and the Ethics Committee of Luohe Central Hospital. Participants received detailed information on the study’s purpose, significance, and risks before participating, in accordance with the Declaration of Helsinki. Written informed consent was obtained from all participants. The inclusion criteria for this study are as follows: (1) age: 35–75 years (born between 1 January 1946 and 31 December 1986), (2) residing near the survey site for at least 6 months in the 12 months prior to screening, and (3) voluntarily participating in this project and signing the informed consent form. The exclusion criteria are as follows: (1) survey participants with missing data, (2) survey participants who answered “unclear” to questionnaire items, and (3) survey participants with fasting time less than 8h before blood collection.

We utilized standardized questionnaires to collect data on sociodemographic characteristics (e.g., age, gender), lifestyle factors (e.g., smoking, alcohol consumption), medical history (including hypertension, diabetes, dyslipidemia, coronary heart disease, myocardial infarction, stroke), and medication usage (such as antihypertensive, hypoglycemic, and lipid-lowering drugs) of the study participants. Data were collected through an information system provided by the ChinaHEART study and were uploaded directly to the national study center in the Fuwai Hospital. The anonymous data used in the current study were applied from the national study center in the Fuwai Hospital after they performed data washing. CVD was defined as a diagnosis of coronary heart disease, previous myocardial infarction, or stroke. Body mass index (BMI) was calculated as body mass in kilograms divided by the square of height in meters. Waist circumference was measured by placing a measuring tape horizontally around the abdomen at the midpoint between the anterior superior iliac crest and the lower edge of the twelfth rib. Systolic blood pressure (SBP) and diastolic blood pressure (DBP) were measured in a seated resting position at least twice, and the average values were recorded. For lipid profiles, fingertip blood samples were collected after an overnight fast of at least 8h and assessed accordingly. However, 267 participants (3.9%) were excluded due to irreversible missing lipid profile data (invalid fingertip blood samples that could not be rectified), leaving 6,593 participants for final analysis.

According to the risk assessment prediction map in the Guidelines for Cardiovascular Risk Assessment and Management issued by the World Health Organization in 2008, specifically the WHO/ISH Risk Prediction Charts for the Western Pacific Region B, the risk assessment of CVD was conducted (11). If the screening subjects had a 10-year risk of CVD of 20%, they were judged as high risk.

### Statistics

The study population’s basic characteristics were categorized by quartiles of the TG/HDL-C ratio for descriptive analysis. Normality of continuous variables was assessed using the Kolmogorov–Smirnov test, with mean ± standard deviation for normally distributed data and median (25%–75% interval) for non-normally distributed data. Categorical variables were described using percentages. Distribution differences in CVD high-risk proportions across TG/HDL-C ratio quartile populations were illustrated with a percentage stacking bar graph. Continuous variable comparisons between groups involved t-tests or analysis of variance for normally distributed data and rank sum tests for non-normally distributed data. Chi-square tests were conducted for categorical variable comparisons. The association between the TG/HDL-C ratio and CVD high risk was examined using logistic regression analysis, with two models utilized: one without confounder adjustments for univariate analysis and another adjusting for confounding factors including gender, age, marital status, BMI, waist circumference, fasting blood glucose, smoking history, alcohol consumption history, diabetes, and systolic and DBP. Nonlinear associations between the TG/HDL-C ratio and CVD high risk were explored through restricted cubic spline analysis, utilizing key points at the 10th, 25th, 50th, 75th, and 90th percentiles of the TG/HDL-C ratio. The ability of the TG/HDL-C ratio to identify CVD high-risk was evaluated using receiver operating characteristic (ROC) curve analysis, with a 1,000-iteration bootstrap to correct for optimism bias. The optimal cutoff was determined using the Youden index (sensitivity + specificity - 1), distinguishing participants into low and high TG/HDL-C groups. Furthermore, we used the net reclassification improvement (NRI) index to quantify the added value of TG/HDL-C for the discriminative performance.

## Results

**Figure 1:**
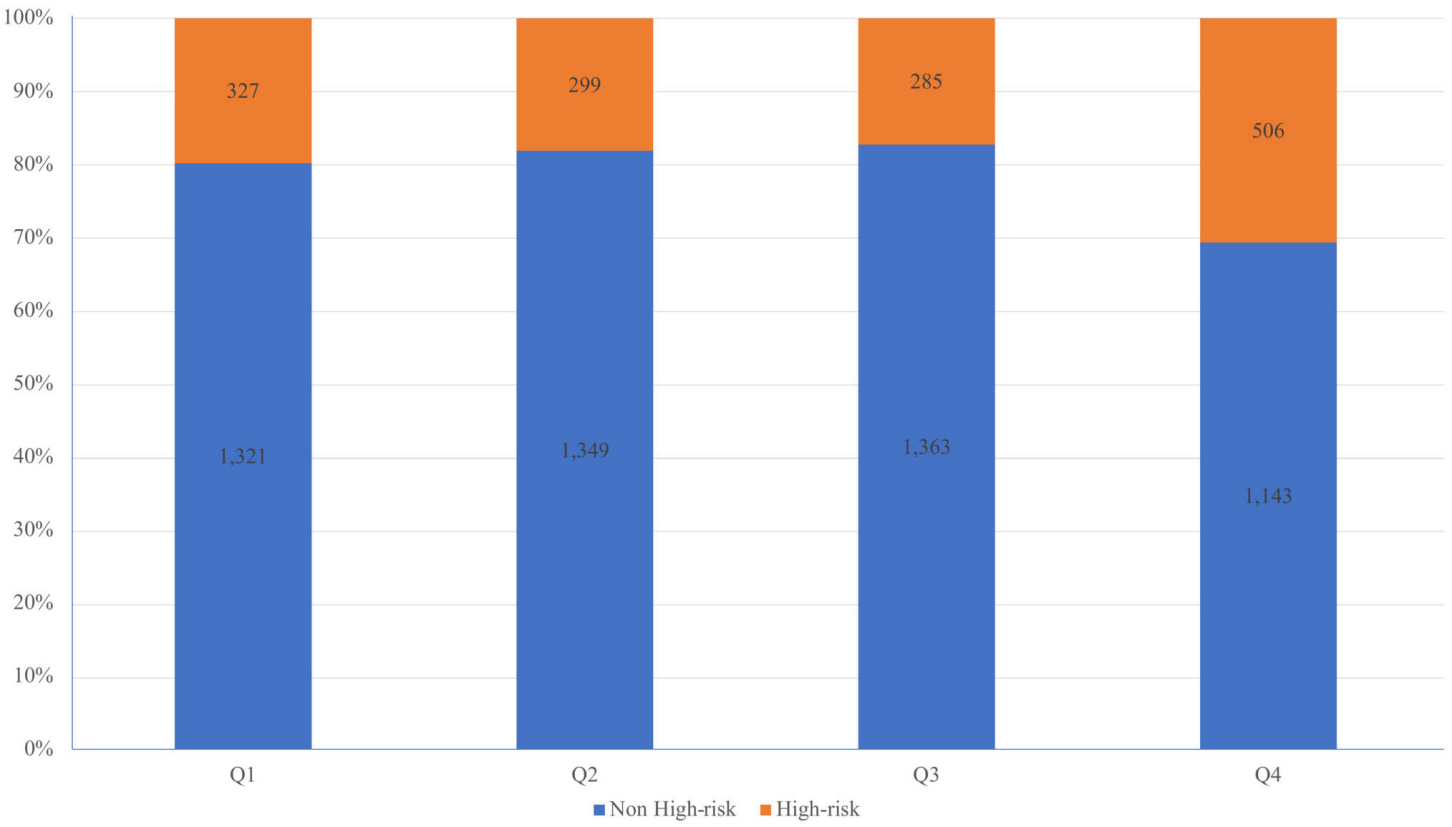
Percentage stacked histogram to visually represent the distribution of CVD high-risk individuals across quartiles of the TG/HDL-C ratio.

Within the study, a total of 6,593 participants with sufficient data were included, consisting of 1,417 individuals categorized as CVD high-risk and 5,176 individuals in the non-high-risk category. The study cohort was stratified by quartiles of the TG/HDL-C ratio, highlighting notable variations in demographic and clinical characteristics such as age, gender, height, weight, BMI, waist circumference, smoking habits, alcohol consumption, and history of hypertension and diabetes, as well as levels of glucose, lipids, and blood pressure (refer to **Table 1**). The distribution of high-risk individuals across these quartiles was 20%, 18%, 17%, and 31%, respectively (view **Figure 1**).

**Table 1:** Clinical characteristics of the study population by quartiles of TG/HDL-C.

Characteristics	Q1	Q2	Q3	Q4	*P*-value
Number	*N* = 1,648	*N* = 1,648	*N* = 1,648	*N* = 1,649	
Age [median (IQR)]	59.00 (50.00–67.00)	58.00 (50.00–67.00)	58.00 (51.00-66.00)	58.00 (52.00–66.00)	0.780
Male (%)	617 (37%)	613 (37%)	599 (36%)	667 (40%)	0.081
BMI [median (IQR)]	24.12 (22.14–26.32)	24.80 (22.70–26.83)	25.15 (23.16–27.44)	26.04 (24.01–28.36)	< 0.001
Waist [median (IQR)]	83.00 (77.00–89.95)	85.00 (79.00–91.00)	86.00 (80.50–93.00)	89.00 (82.00–95.00)	< 0.001
SBP [median (IQR)]	135.00 (123.25–148.50)	136.00 (124.50–149.50)	136.00 (125.50–149.50)	141.50 (129.50–152.00)	< 0.001
DBP [median (IQR)]	82.50 (76.00–89.50)	82.50 (76.00–89.50)	82.50 (76.00–90.00)	85.00 (77.00–92.50)	< 0.001
Current smoke (%)	338 (21%)	325 (20%)	300 (18%)	358 (22%)	0.083
Drinking (%)	86 (5%)	68 (4%)	98 (6%)	107 (6%)	0.018
HTN (%)	335 (20%)	338 (21%)	367 (22%)	467 (28%)	< 0.001
DM (%)	72 (4%)	96 (6%)	91 (6%)	147 (9%)	< 0.001
CHD (%)	36 (2%)	25 (2%)	19 (1%)	13 (1%)	0.006
Stroke (%)	63 (4%)	37 (2%)	41 (2%)	40 (2%)	0.021
TC [median (IQR)]	4.08 (3.47–4.65)	4.53 (4.03–5.11)	4.97 (4.36–5.43)	5.62 (4.98–6.39)	< 0.001
HDL [median (IQR)]	1.71 (1.49–1.97)	1.50 (1.33–1.69)	1.39 (1.23–1.52)	1.21 (1.09–1.37)	< 0.001
TG [median (IQR)]	1.21 (0.95–1.62)	1.41 (1.11–1.74)	1.58 (1.25–2.00)	1.94 (1.47–2.69)	< 0.001
LDL [median (IQR)]	1.96 (1.45–2.44)	2.48 (2.09–2.88)	2.71 (2.32–3.17)	3.35 (2.71–4.03)	< 0.001
Glu [median (IQR)]	5.40 (5.10–5.80)	5.40 (5.10–5.90)	5.40 (5.11–6.00)	5.70 (5.30–6.40)	< 0.001
TG/HDL-C [median (IQR)]	2.43 (2.20–2.59)	3.02 (2.88–3.15)	3.55 (3.41-3.72)	4.50 (4.17–4.97)	< 0.001
CVD high risk (%)	257 (16%)	251 (15%)	290 (18%)	620 (38%)	< 0.001

Data are presented as median (IQR) for continuous variables and number (percentage) for categorical variables. HTN, hypertension; DM, diabetes mellitus; CHD, coronary heart disease; BMI, body mass index; TC, total cholesterol; TG, triglyceride; LDL, low density lipoprotein; HDL, high density lipoprotein; SBP, systolic blood pressure; DBP, diastolic blood pressure.

**Figure 2:**
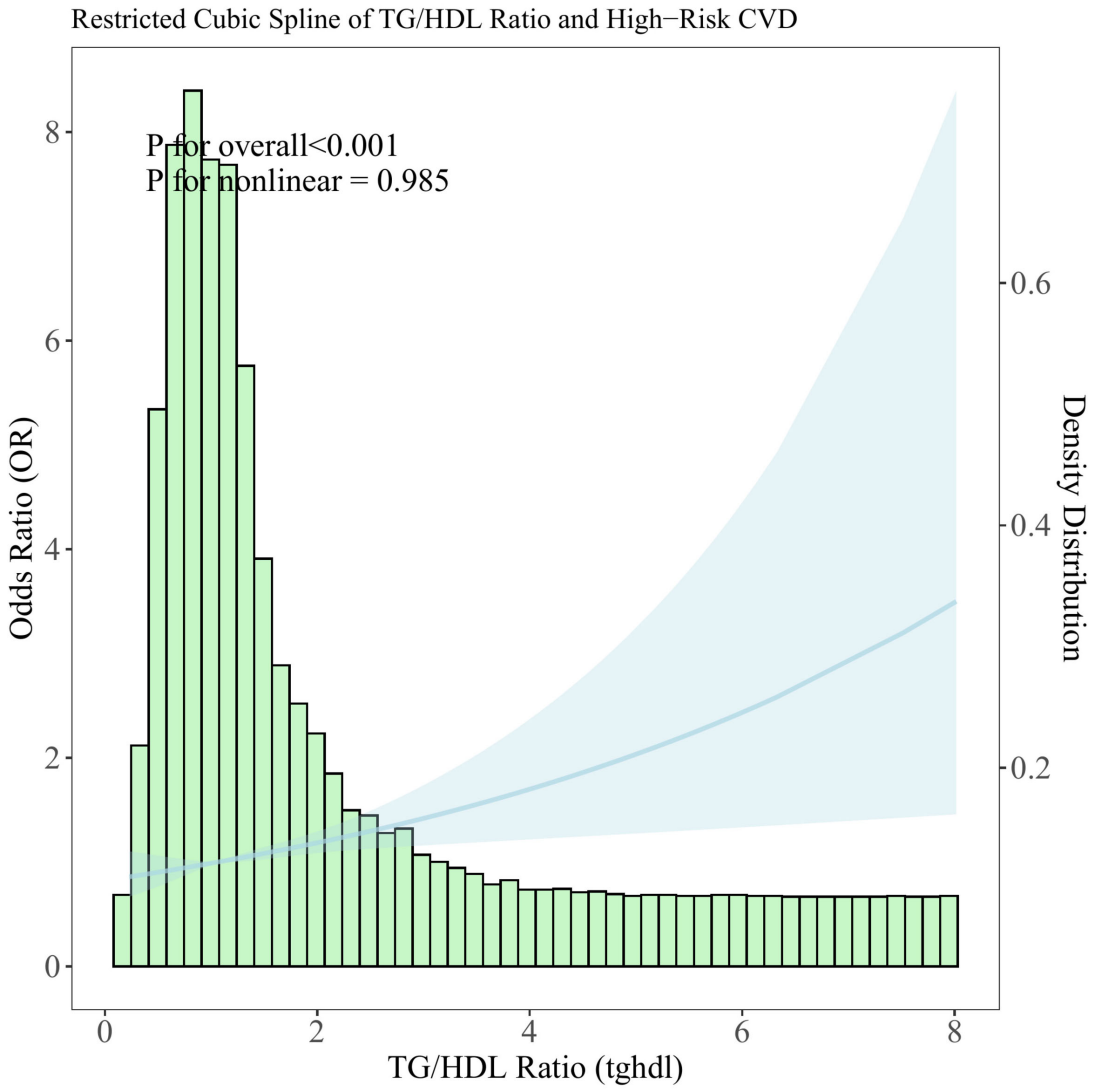
Distribution histogram of the TG/HDL-C ratio in the study population and restricted cubic spline to assess the relationship between TG/HDL-C ratio and CVD high risk.

To explore the nonlinear relationship between the TG/HDL-C ratio and CVD high-risk, a restricted cubic spline analysis was conducted. The analysis revealed a positive linear association between elevated TG/HDL-C ratio levels and an increased likelihood of high-risk CVD (see **Figure 2**).

**Figure 3:**
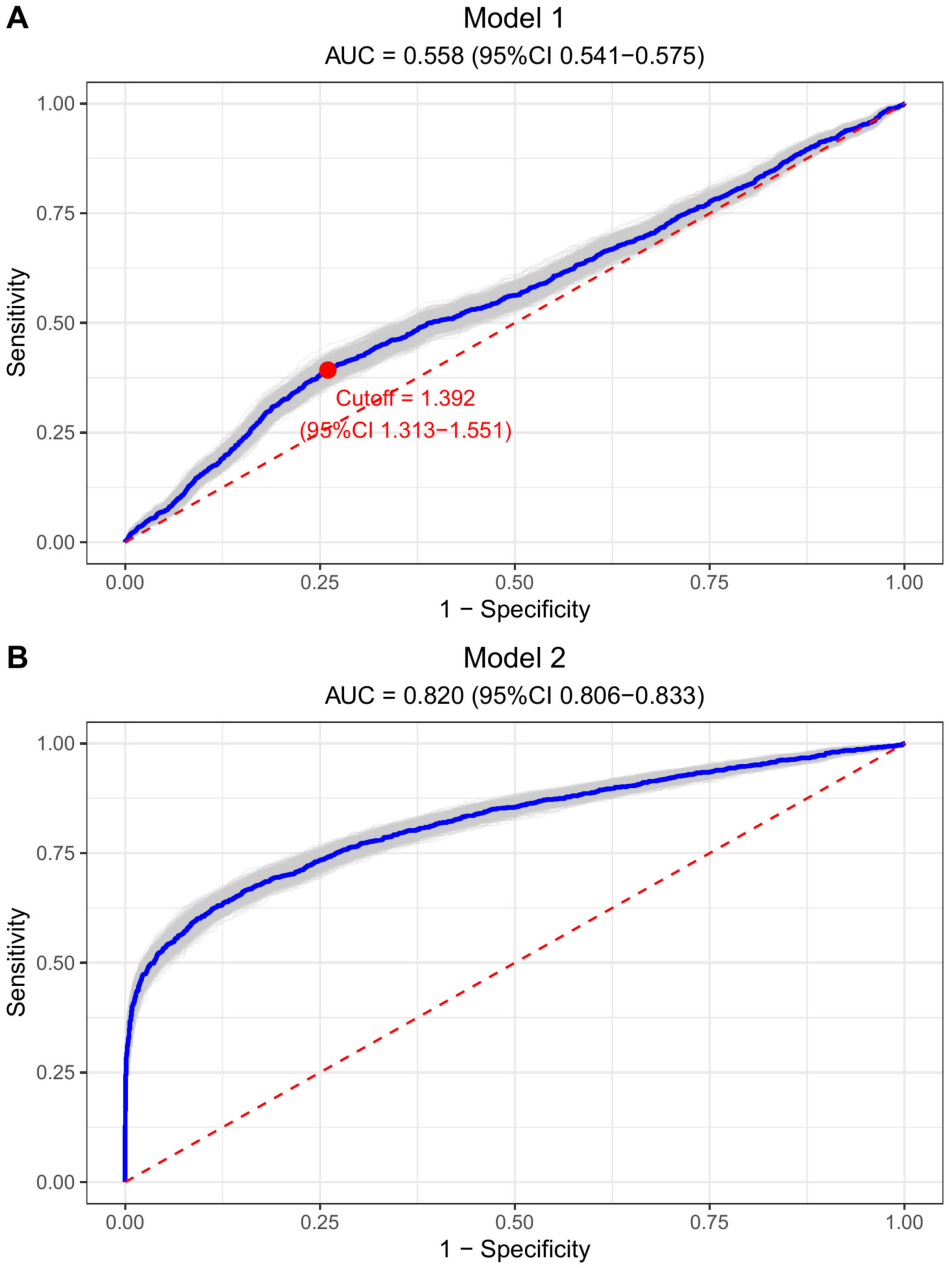
Bootstrap ROC curves to illustrate the discriminatory power of TG/HDL-C ratio for CVD high risk. **(A)** Model 1 (TG/HDL-C ratio alone): AUC = 0.558, optimal cutoff = 1.392. **(B)** Model 2 (fully adjusted): AUC = 0.820. Both plots show sensitivity vs. 1-specificity with diagonal reference line.

The TG/HDL-C ratio’s discriminatory ability in identifying individuals at CVD high-risk was evaluated through ROC analysis (refer to **Figure 3**). Initially, in Model 1, the AUC for the TG/HDL-C ratio alone was 0.558 (95% CI: 0.541–0.575); upon incorporating all covariates into the analysis in model 2, the AUC significantly increased to 0.820 (95% CI: 0.806–0.833), suggesting the enhanced effectiveness of combining the TG/HDL-C ratio with traditional cardiovascular risk factors for high-risk identification. Notably, the NRI index showed a significant improvement in discriminative performance, with a value of 0.107 ± 0.030 (*P* = 0.016). Moreover, the ROC analysis identified the optimal cutoff value for the TG/HDL-C ratio as 1.392 (1.313–1.551), enabling the classification of participants into high and low TG/HDL-C ratio groups (set at 1.40 for convenience).

The logistic regression analysis results examining the relationship between the TG/HDL-C ratio and CVD high-risk are detailed in **Table 2**. In Model 1, a one-unit increase in the TG/HDL-C ratio was associated with a 1.40-fold increase in the odds of CVD high-risk (95% CI: 1.30–1.51, *P* < 0.001). Comparatively, the high TG/HDL-C ratio group exhibited 1.83 (1.62–2.07, *P* < 0.001) times higher odds of CVD high-risk than the low TG/HDL-C ratio group, while the highest quartile of the TG/HDL-C ratio had 1.79 times higher odds compared to the lowest quartile (95% CI: 1.52–2.10, *P* < 0.001). In Model 2, each one-unit rise in the TG/HDL-C ratio was associated with a 1.21-fold increase in the odds of CVD high-risk (95% CI: 1.11–1.32, *P* < 0.001). Additionally, the high TG/HDL-C ratio group demonstrated 1.34 (1.15–1.56, *P* < 0.001) times higher odds of CVD high-risk compared to the low TG/HDL-C ratio group, with the highest quartile of the TG/HDL-C ratio displaying 1.49 times higher odds than the lowest quartile (95% CI: 1.21–1.83, *P* < 0.001).

**Table 2:** Association between TG/HDL-C ratio and CVD high-risk by logistic regression.

Groups	Model 1		Model 2	
	OR (95% CI)	*P*	OR (95% CI)	*P*
All participants				
TG/HDL-C (per 1 unit)	1.40 (1.30–1.51)	< 0.001	1.21 (1.11–1.32)	< 0.001
TG/HDL-C (high vs. low)	1.83 (1.62–2.07)	< 0.001	1.34 (1.15–1.56)	< 0.001
TG/HDL-C (quartile)				
Q1	Ref		Ref	
Q2	0.90 (0.75–1.07)	0.214	1.04 (0.85–1.29)	0.68
Q3	0.84 (0.71–1.01)	0.06	0.95 (0.77–1.18)	0.634
Q4	1.79 (1.52–2.10)	< 0.001	1.49 (1.21–1.83)	< 0.001
Female				
TG/HDL-C (per 1 unit)	1.36 (1.24–1.50)	< 0.001	1.14 (1.01–1.28)	0.028
TG/HDL-C (high vs. low)	1.52 (1.30–1.77)	< 0.001	1.24 (1.03–1.50)	0.026
TG/HDL-C (quartile)				
Q1	Ref		Ref	
Q2	0.94 (0.74–1.19)	0.6	0.97 (0.74–1.28)	0.838
Q3	0.91 (0.72–1.15)	0.447	1.00 (0.76–1.32)	0.998
Q4	1.78 (1.44–2.20)	< 0.001	1.33 (1.02–1.74)	0.034
Male				
TG/HDL-C (per 1 unit)	1.50 (1.33–1.69)	< 0.001	1.31 (1.14–1.52)	< 0.001
TG/HDL-C (high vs. low)	2.08 (1.71–2.53)	< 0.001	1.47 (1.15–1.88)	0.002
TG/HDL-C (quartile)				
Q1	Ref		Ref	
Q2	0.85 (0.66–1.11)	0.238	1.14 (0.84–1.57)	0.432
Q3	0.78 (0.59–1.03)	0.076	0.90 (0.64–1.27)	0.557
Q4	1.94 (1.51–2.49)	< 0.001	1.67 (1.20–2.32)	0.002

## Discussion

CVD remains a significant global health burden, necessitating the identification of individuals at high risk for effective prevention and intervention strategies. The TG/HDL-C ratio has emerged as a potential marker for assessing CVD risk in the general population. It has been observed that the TG/HDL-C ratio not only provides an approximation of insulin resistance but also assists in identifying patients with an atherogenic lipoprotein profile (12). The typical lipoprotein phenotype in insulin-resistant/hyperinsulinemic patients is typified by high plasma TG and low HDL cholesterol levels, smaller and denser LDL particles, as well as an increased accumulation of remnant lipoproteins after a meal. This lipoprotein phenotype is not only associated with insulin resistance, but it is also linked with an elevated risk of CVD (13). Although plasma TG and HDL cholesterol levels are commonly tested, the same cannot be said for the LDL particle diameter or postprandial remnant concentrations. Nonetheless, a previous study observed a robust correlation between the TG/HDL cholesterol ratio and the LDL peak diameter, suggesting its potential as an indirect marker of small dense LDL accumulation (LDL phenotype B) (14). Moreover, it is suggested that a TG/HDL cholesterol concentration ratio of 3.5 can accurately predict the presence of this phenotype, which is associated with a higher risk of CVD. Therefore, by assessing plasma TG and HDL cholesterol levels and computing their ratio, it is possible to obtain understanding into three out of the four modifications in lipoprotein metabolism that enhance the risk of CVD in insulin-resistant patients.

Numerous epidemiological studies have examined the relationship between the TG/HDL ratio and CVD risk. Most of these studies have reported a positive association between a higher TG/HDL ratio and increased CVD risk. For instance, a prospective cohort study by Gaziano et al. found that the ratio of triglycerides to HDL was a strong predictor of myocardial infarction (15); Urbina et al. suggest that the TG/HDL ratio may be a useful marker for identifying young individuals at risk for developing arterial stiffness, an early sign of CVD (16); Li et al. found a positive correlation between the TG/HDL-C ratio and carotid intima thickness, a marker of early atherosclerosis, in adolescents with type 2 diabetes (17); Du et al. found that the TG/HDL ratio is associated with insulin resistance, a major risk factor for CVD, in a Chinese population (18); Ren et al. found that the TG/HDL ratio is positively associated with insulin resistance in newly diagnosed type 2 diabetes patients, indicating an increased risk of CVD (19); Squillace et al. found that a higher TG/HDL ratio is predictive of the development of diabetes, a major risk factor for CVD (20); Hong et al. found that a high TG/HDL ratio is associated with an increased risk of cardiovascular events in patients with diabetes and coronary artery disease (21).

Notably, recent evidence further supports the role of TG/HDL-C in early vascular pathological changes. Di Marco et al. demonstrated that in prediabetic populations, the TG/HDL-C ratio, rather than the triglyceride-glucose (TyG) index, is independently associated with early arterial stiffness (22). This finding aligns with our results and reinforces that the TG/HDL-C ratio serves as a sensitive surrogate marker for early vascular remodeling. Mechanistically, this association may be driven by the interplay between TG/HDL-C imbalance and key pathological processes: elevated TG levels promote the formation of triglyceride-rich lipoproteins and their remnants, which induce endothelial dysfunction by increasing oxidative stress and proinflammatory cytokine release; conversely, reduced HDL-C impairs its reverse cholesterol transport function and antioxidant capacity, further exacerbating vascular injury. Additionally, the TG/HDL-C ratio reflects underlying insulin resistance, which disrupts lipoprotein metabolism (e.g., increasing small dense LDL particles) (23) and accelerates atherogenesis, linking metabolic derangements to vascular damage in a coherent pathological pathway (24).

The TG/HDL ratio holds clinical significance as a potential marker for CVD prevention. It provides valuable insights into the balance between TG and HDL levels, representing the interplay between dyslipidemia and atherosclerosis. A higher TG/HDL ratio reflects an increased atherogenic lipid profile and has been associated with arterial stiffness, carotid intima thickness, insulin resistance, and diabetes, all of which contribute to CVD development (14, 25). By incorporating the TG/HDL ratio into risk assessment models, clinicians can more accurately identify individuals at high risk for CVD and implement targeted interventions (26, 27).

In the context of precision cardiometabolic medicine, the TG/HDL-C ratio offers unique value beyond traditional risk stratification: it can serve as a “metabolic signature” to guide personalized preventive strategies. For example, individuals with a higher TG/HDL-C ratio may benefit from early, targeted interventions—such as lifestyle modifications (e.g., low-sugar and low-saturated-fat diets, regular aerobic exercise) to improve lipid metabolism, or pharmacological interventions in high-risk subgroups. Importantly, the evolving landscape of lipid-lowering therapies further highlights the clinical relevance of the TG/HDL-C ratio. According to the review by Di Giacomo-Barbagallo et al., emerging RNA-based therapies like inclisiran not only achieve sustained LDL-C reduction but also exert indirect effects on TG metabolism and HDL function in some patients (28). Integrating the TG/HDL-C ratio into treatment monitoring could help evaluate the comprehensive metabolic benefits of such therapies, linking lipid modulation to inflammation control and vascular protection—thus advancing the goal of precision CVD prevention.

Despite the clinical significance of the TG/HDL ratio, several limitations must be acknowledged. First, the study’s cross-sectional design cannot establish temporal or causal relationships between the TG/HDL-C ratio and CVD risk, as it only captures a single-timepoint snapshot of exposure and outcome. Second, CVD high-risk was defined using WHO risk charts rather than hard clinical outcomes (e.g., myocardial infarction, stroke); this reliance on estimated risk scores introduces subjectivity and may overstate predictive accuracy. Third, cut-off values for high TG/HDL ratios vary across studies and thus may not generalize to international populations, and lack of global standardization limits cross-study comparability. Fourth, confounding factors (age, sex, lifestyle, comorbidities) may influence the observed association; though adjusted for, the fully adjusted model’s AUC of 0.820 may be inflated due to conceptual overlap between some covariates (e.g., BMI, fasting glucose) and both the TG/HDL-C ratio (exposure) and CVD risk (outcome), creating circularity. Fifth, the study population was exclusively from Luohe in Central China, restricting generalizability to other regions, ethnic groups, or healthcare contexts. Sixth, lipid measurements relied on fingertip blood instead of standardized venous samples, potentially introducing measurement variability; additionally, variations in laboratory lipid assay methods may further affect the accuracy of TG and HDL cholesterol estimations.

In conclusion, the TG/HDL ratio shows promise for CVD risk assessment, as it captures the interplay between triglycerides and HDL cholesterol to reflect the atherogenic lipid profile. Incorporating it into risk assessment models may provide additional information to healthcare professionals for risk stratification and identifying individuals who could benefit from closer monitoring or early interventions. Moving forward, longitudinal validation with hard clinical outcomes, standardization of cutoff values, stricter control of confounding factors, and harmonization of laboratory methods are needed to further evaluate its potential. With such evidence, the TG/HDL ratio may eventually contribute to refined CVD prevention efforts.

## Data Availability

The raw data supporting the conclusions of this article will be made available by the authors, without undue reservation.
